# HIV-Infected Macrophages Are Infected and Killed by the Interferon-Sensitive Rhabdovirus MG1

**DOI:** 10.1128/JVI.01953-20

**Published:** 2021-04-12

**Authors:** Teslin S. Sandstrom, Nischal Ranganath, Stephanie C. Burke Schinkel, Syim Salahuddin, Oussama Meziane, Sandra C. Côté, Cecilia T. Costiniuk, Mohammad-Ali Jenabian, Jonathan B. Angel

**Affiliations:** a Department of Biochemistry, Microbiology & Immunology, University of Ottawa, Ottawa, Ontario, Canada; b Ottawa Hospital Research Institute, Ottawa, Ontario, Canada; c Chronic Viral Illness Service and Division of Infectious Diseases, Department of Medicine, McGill University Health Centre, Montreal, Quebec, Canada; d Infectious Diseases and Immunity in Global Health Program, Research Institute of McGill University Health Centre, Montreal, Quebec, Canada; e Department of Biological Sciences, University of Quebec at Montreal (UQAM), Montreal, Quebec, Canada; f Department of Microbiology and Immunology, McGill University, Montreal, Quebec, Canada; g Department of Microbiology, Infectiology and Immunology, Faculty of Medicine, University of Montreal, Montreal, Quebec, Canada; h Division of Infectious Diseases, Ottawa Hospital–General Campus, Ottawa, Ontario, Canada; Emory University

**Keywords:** HIV, interferon, MG1, macrophage, oncolytic virus

## Abstract

The use of unique cell surface markers to target and eradicate HIV-infected cells has been a longstanding objective of HIV-1 cure research. This approach, however, overlooks the possibility that intracellular changes present within HIV-infected cells may serve as valuable therapeutic targets. For example, the identification of dysregulated antiviral signaling in cancer has led to the characterization of oncolytic viruses capable of preferentially killing cancer cells. Since impairment of cellular antiviral machinery has been proposed as a mechanism by which HIV-1 evades immune clearance, we hypothesized that HIV-infected macrophages (an important viral reservoir *in vivo*) would be preferentially killed by the interferon-sensitive oncolytic Maraba virus MG1. We first showed that HIV-infected monocyte-derived macrophages (MDM) were more susceptible to MG1 infection and killing than HIV-uninfected cells. As MG1 is highly sensitive to type I interferons (IFN-I), we then investigated whether we could identify IFN-I signaling differences between HIV-infected and uninfected MDM and found evidence of impaired IFN-α responsiveness within HIV-infected cells. Finally, to assess whether MG1 could target a relevant, primary cell reservoir of HIV-1, we investigated its effects in alveolar macrophages (AM) obtained from effectively treated individuals living with HIV-1. As observed with *in vitro*-infected MDM, we found that HIV-infected AM were preferentially eliminated by MG1. In summary, the oncolytic rhabdovirus MG1 appears to preferentially target and kill HIV-infected cells via impairment of antiviral signaling pathways and may therefore provide a novel approach to an HIV-1 cure.

**IMPORTANCE** Human immunodeficiency virus type 1 (HIV-1) remains a treatable, but incurable, viral infection. The establishment of viral reservoirs containing latently infected cells remains the main obstacle in the search for a cure. Cure research has also focused on only one cellular target of HIV-1 (the CD4^+^ T cell) while largely overlooking others (such as macrophages) that contribute to HIV-1 persistence. In this study, we address these challenges by describing a potential strategy for the eradication of HIV-infected macrophages. Specifically, we show that an engineered rhabdovirus*—*initially developed as a cancer therapy*—*is capable of preferential infection and killing of HIV-infected macrophages, possibly via the same altered antiviral signaling seen in cancer cells. As this rhabdovirus is currently being explored in phase I/II clinical trials, there is potential for this approach to be readily adapted for use within the HIV-1 cure field.

## Abstract (precis)

Human immunodeficiency virus type 1 (HIV-1) remains a treatable, but incurable, viral infection. The establishment of viral reservoirs containing latently infected cells remains the main obstacle in the search for a cure.

## INTRODUCTION

Human immunodeficiency virus type 1 (HIV-1) cure research has largely concentrated on the eradication of latently infected CD4^+^ T cells, while overlooking other cellular constituents of the viral reservoir. Tissue-resident macrophages, for example, contribute to HIV-1 persistence but remain poorly characterized (1–5). Although strategies by which to target these cells have been proposed (6–8), the development of a cure cannot proceed without a better understanding of the macrophage HIV-1 reservoir.

It has been well established that the ability of HIV-1 to infect and replicate within susceptible target cells is due, in part, to its ability to shut down antiviral cellular signaling pathways. As evidence of this, the degradation of key RNA-sensing proteins (9), downregulation of key antiviral interferon (IFN)-stimulated genes (ISG) (10, 11), and impaired release of type I IFN (IFN-I) (12–14), have all been observed within HIV-infected macrophages. In addition to its antiviral effects, impairment of the IFN-I response permits rapid cellular proliferation and survival (15) and is therefore a common feature of malignant cells. This observation has led to the development of therapeutic oncolytic viruses (OV) that are genetically engineered to be highly sensitive to IFN-I and can therefore be used to preferentially eradicate cancer cells while sparing healthy, nonmalignant cells (16, 17).

Given the parallels between malignant and HIV-infected cells, we questioned whether one such OV, the recombinant Maraba virus MG1, could be employed in the context of HIV-1 infection. We have previously shown that both HIV-infected cell lines and primary CD4^+^ T cells were preferentially infected and killed by MG1 (18, 19), suggesting that MG1 has therapeutic potential for the preferential eradication of the HIV-1 reservoir. We therefore hypothesized that HIV-infected, primary monocyte-derived macrophages (MDM) would also be preferentially infected and killed by MG1.

We first demonstrated the susceptibility of HIV-infected MDM to MG1-mediated killing by measuring markers of cell death and proviral HIV-1 DNA following OV infection. Cell death was not observed after exposure to UV-inactivated MG1, or to filtered supernatants from MG1-infected MDM, confirming that replication-competent MG1 was responsible for the preferential killing of HIV-infected cells. Because MG1 is highly sensitive to IFN-I, we questioned whether HIV-infected MDM possessed changes in IFN-I signaling that were not present in HIV-uninfected cells. Basal levels of two representative ISG were higher in HIV-infected MDM, but IFN-α-induced ISG expression in these cells was impaired, suggesting that alterations in antiviral signaling may be responsible for increased MG1-mediated cytopathogenicity. Finally, we demonstrated the ability of MG1 to eliminate HIV-infected alveolar macrophages (AM) from combination antiretroviral therapy (cART)-treated people living with HIV-1 (PLWHIV), which represent the only readily accessible source of tissue-resident myeloid cells implicated in HIV-1 persistence (2, 20–22).

## RESULTS

### HIV-infected monocyte-derived macrophages are preferentially infected by MG1.

We have previously shown that the oncolytic Maraba virus MG1 preferentially kills latently HIV-infected primary CD4^+^ T cells (19). Therefore, we questioned whether HIV-infected MDM could also be preferentially infected by MG1.

**FIG 1:**
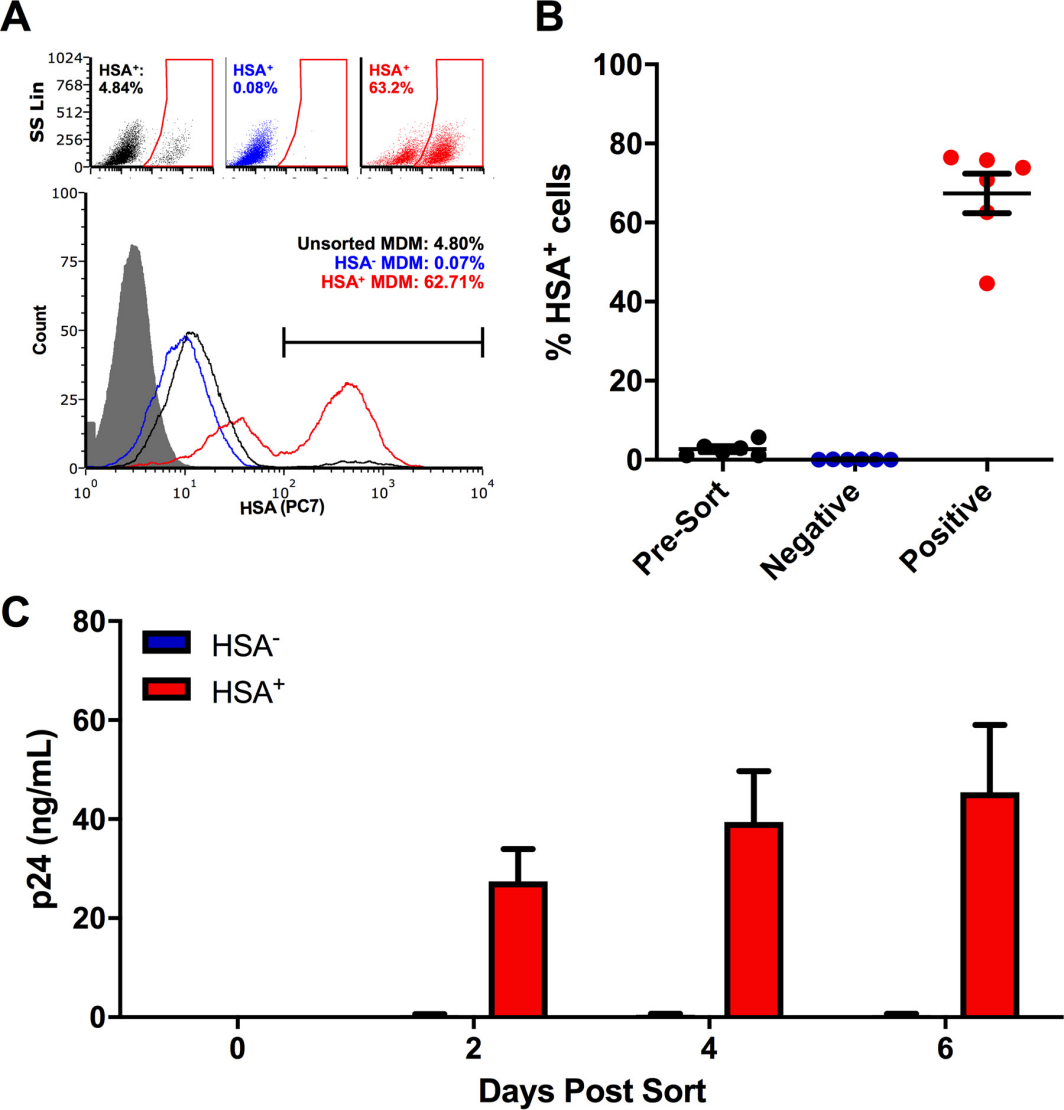
Enrichment of HSA^+^ MDM. (A) Representative dot plots and histogram showing the frequency of HSA^+^ MDM presort (black), as well as within the HSA-negative (blue) and HSA-enriched (red) cell fractions. The HSA isotype control is shown in gray. Histogram peak counts (*y* axis) were normalized to that of the isotype control for visualization purposes. SS Lin, side scatter, linear scale. (B) Purity of HSA-enriched MDM fraction postsort (*n* = 6). (C) HIV-1 p24 antigen release by enriched HSA^+^ and HSA^−^ MDM (*n* = 4). Data represent mean ± standard error of the mean (SEM); *n* values represent separate biological replicates.

First, we established a model of HIV-1 infection in human MDM that allowed us to reliably differentiate between HIV-infected and HIV-uninfected bystander cells. Studies investigating HIV-1 infection of MDM cultures typically obtain results using bulk cell analysis, overlooking the fact that rates of infection in these cells are typically quite low (23, 24). To avoid this, we used the CCR5-tropic reporter virus HIV NL4.3 BAL-IRES-HSA. This virus has been engineered to express murine heat-stable antigen (HSA) (24), which localizes to the cell surface during productive HIV-1 infection and can be used to differentiate infected from bystander (uninfected) MDM. By 6 days postinfection (dpi), distinct HSA-positive (HSA^+^) and -negative (HSA^−^) populations could be detected by flow cytometry (Fig. 1A). Similar to others (25), we found that HSA^+^ MDM enriched by magnetic bead isolation continued to release HIV-1 p24 antigen into culture supernatants, while HSA^−^ cells did not (Fig. 1B and C). Thus, HSA^+^ MDM were defined as HIV infected and HSA^−^ MDM were defined as bystander populations.

**FIG 2:**
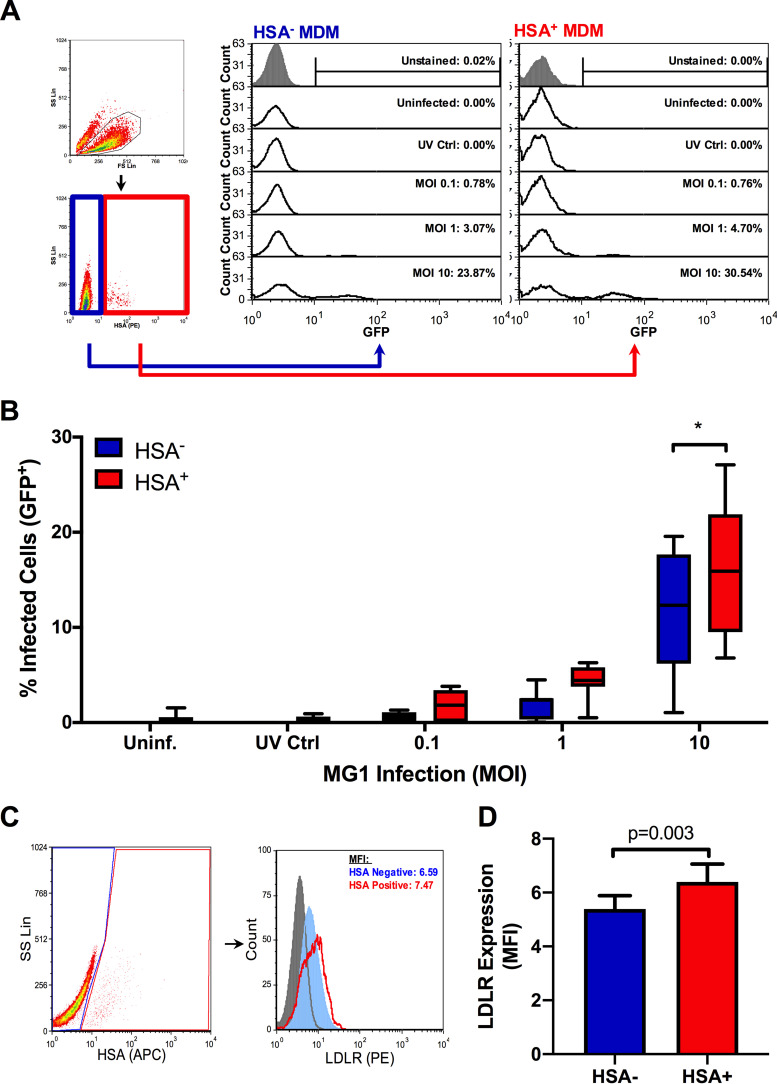
MG1 preferentially infects HSA^+^ MDM. At 6 days post-HIV-1 infection, MDM cultures were infected with MG1 or left uninfected. UV-inactivated MG1 was included as an additional experimental condition (UV Ctrl.). UV-inactivated viral particles were added to MDM cultures at a ratio of 10:1, as calculated from pre-UV inactivation virus titers. At 48 h post-OV infection, frequencies of HSA^+^ and GFP^+^ MDM were measured by flow cytometry. (A) Example of gating strategy employed during data analysis. Intact cells were analyzed (black gate), after which HSA^−^ (blue gate) and HSA^+^ (red gate) MDM were gated upon. The percentages of GFP^+^ cells were then measured within HSA^+^ and HSA^−^ populations, as shown in representative histograms. Histogram peak counts (*y* axis) for HSA^−^ and HSA^+^ populations were normalized to that of the uninfected control for visualization purposes. (B) Frequencies of GFP^+^ cells within HSA^+^ and HSA^−^ MDM populations at 48 h post-MG1 infection (*n* = 7; *P* < 0.0001 by 2-way repeated-measures ANOVA; *, *P* < 0.001 by Bonferroni posttest). (C) Flow cytometry gating strategy and representative histograms depicting LDLR expression on HSA^−^ (blue) and HSA^+^ (red) MDM. Intact cells were gated (black), after which HSA^−^ (blue) and HSA^+^ (red) MDM were defined. The PE FMO control is shown as a filled, gray peak. Histogram peak counts (*y* axis) for HSA^−^ and HSA^+^ populations were normalized to that of the PE FMO control for visualization purposes. (D) LDLR expression on HSA^−^ (blue) and HSA^+^ (red) MDM (*n* = 7; *P* = 0.003 by paired, two-tailed *t* test). Data represent mean ± SEM; *n* values represent separate biological replicates.

Using this model, bulk MDM cultures consisting of both HSA^+^ and HSA^−^ cells were exposed to the green fluorescent protein (GFP)-expressing MG1 virus. After 48 h, the proportion of GFP-positive cells was assessed by flow cytometry (Fig. 2A, representative histograms). As evidence for the preferential infection of HIV-infected MDM by MG1, a significantly greater percentage of GFP-positive cells was seen in the HSA^+^ MDM population than in the HSA^−^ cells (Fig. 2B). Since this preferential infection could be explained, in part, by elevated surface expression of the MG1 receptor on HIV-infected cells, we measured the expression of the low-density lipoprotein receptor (LDLR) (26–28) and found that its expression was slightly higher on HSA^+^ cells (Fig. 2C and D). Differences in MG1 infection may therefore be partially receptor mediated, although the biological relevance in this small difference in surface expression remains undetermined.

### MG1 preferentially kills HIV-infected monocyte-derived macrophages.

**FIG 3:**
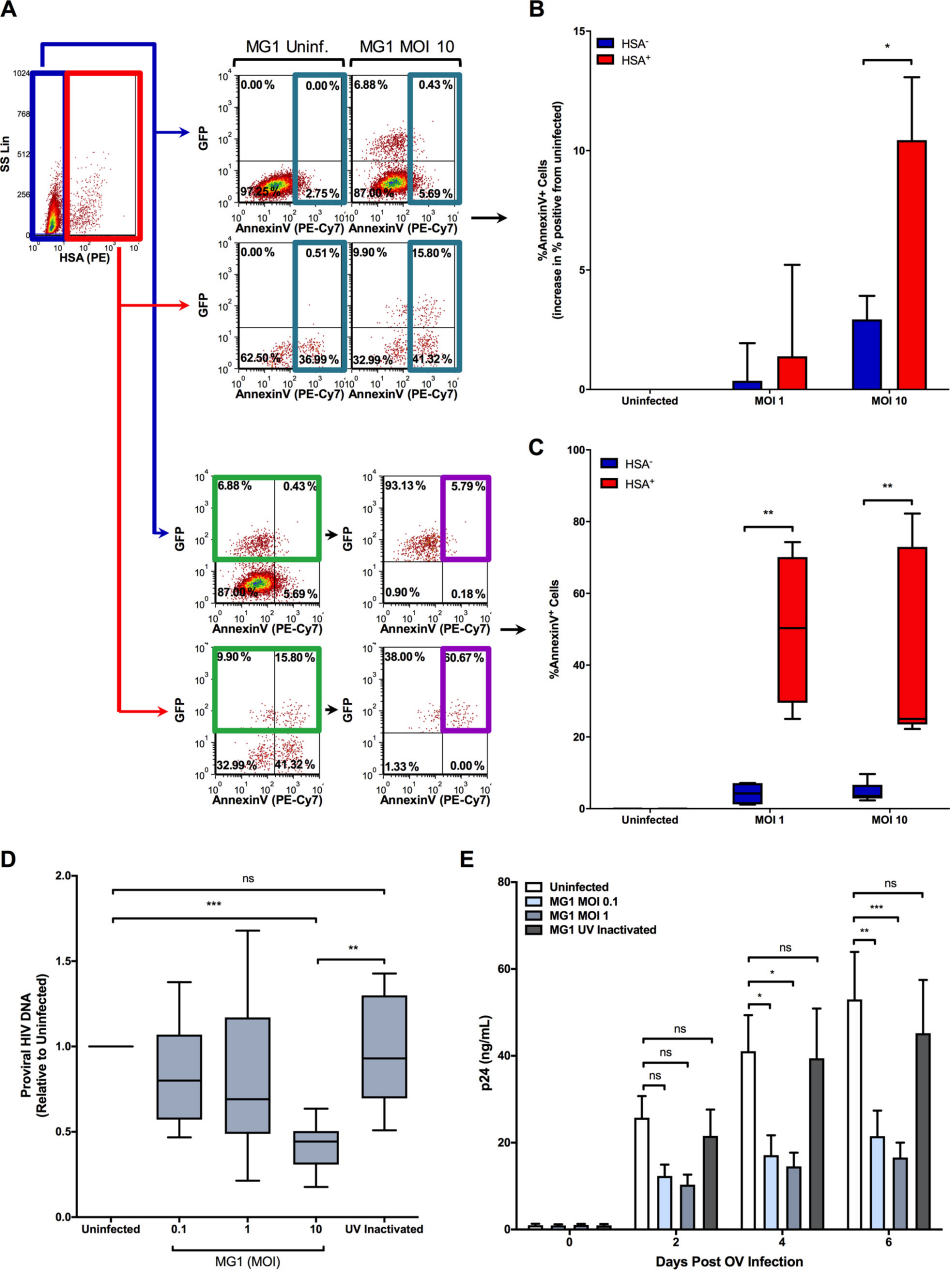
MG1-infected, HSA^+^ MDM preferentially killed by MG1. (A) Representative dot plots depicting the gating strategy used to identify HSA^−^/GFP^+^/annexin V^+^ and HSA^+^/GFP^+^/annexin V^+^ MDM, as shown in panels B and C. HSA^−^ (blue) and HSA^+^ (red) MDM were first defined by flow cytometry. To demonstrate that the surface expression of phosphatidylserine was increased following MG1 infection, the frequency of annexin V^+^ cells within the HSA^−^ or HSA^+^ gates was measured at 24 h post-MG1 infection (top scatterplots; teal gates). To measure the amount of annexin V^+^ cells within the MG1-infected population, GFP^+^ cells were gated upon (bottom; green gate), and the frequency of annexin V^+^ MDM within HSA^+^/GFP^+^ and HSA^−^/GFP^+^ cell gates was measured (bottom; purple gate). (B) Cumulative data showing the difference in frequency of annexin V^+^ cells between MG1-infected and uninfected MDM cultures (*n* = 4; *P* = 0.013 by 2-way repeated-measures ANOVA; *, *P* < 0.05 by Bonferroni posttest). (C) Cumulative data showing the frequency of annexin V^+^ cells within HSA^+^/GFP^+^ and HSA^−^/GFP^+^ MDM gates. This was shown as the percentage of HSA^−^/GFP^+^/annexin V^+^ MDM and HSA^+^/GFP^+^/annexin V^+^ MDM at 24 h post-MG1 infection (uninfected and at an MOI of 10, *n* = 5; at an MOI of 1, *n* = 4; *P* = 0.004 by 2-way repeated-measures ANOVA; **, *P* < 0.01 by Bonferroni posttest). (D) Proviral HIV-1 DNA, measured relative to the MG1-uninfected control, at 48 h post-MG1 infection (*n* = 10; *P* = 0.0004 by 1-way repeated-measures ANOVA; **, *P* < 0.01; ***, *P* < 0.001 by Bonferroni posttest). (E) Quantification of HIV-1 p24 antigen in culture supernatants following MG1 infection (uninfected, MOI 0.1, and MOI 1, *n* = 6; UV inactivated, *n* = 4; *P* < 0.0001 by 2-way ANOVA; *, *P* < 0.05; **, *P* < 0.01; ***, *P* < 0.001 by Bonferroni posttest; ns, not significant). Data represent mean ± SEM; *n* values represent separate biological replicates.

Oncolytic rhabdoviruses, including MG1, have been found to trigger cell death pathways in cancerous cell lines and solid tumors (29–31). To investigate whether preferential infection by MG1 would lead to increased killing of HIV-infected cells, we chose to perform annexin V staining to measure surface levels of phosphatidylserine, an early indicator of apoptosis, on MDM following MG1 infection. First, we assessed whether or not MG1 infection would increase the frequency of annexin V^+^ cells within HSA^+^ and HSA^−^ MDM populations as a whole at 24 h post-MG1 infection. Not only did the frequency of annexin V^+^ cells increase in a multiplicity of infection (MOI)-dependent manner, but this frequency was higher within HSA^+^ MDM than HSA^−^ MDM (Fig. 3A and B). In order to understand whether productive MG1 infection resulted in a difference in annexin V expression between HSA^−^ and HSA^+^ MDM, we next measured annexin V staining on the GFP^+^ cells found within the HSA^+^ and HSA^−^ cell gates and found that GFP^+^/HSA^+^ MDM contained a significantly higher percentage of annexin V^+^ cells than GFP^+^/HSA^−^ MDM (Fig. 3C).

As has been previously demonstrated (19, 32–34), the killing of HIV-infected cells was then confirmed by measuring integrated (proviral) HIV-1 DNA at 2 days post-MG1 infection. Consistent with the annexin V staining, an MOI-dependent decrease in proviral HIV-1 DNA was observed in HIV-infected MDM that had been exposed to infectious MG1 (Fig. 3D). The release of HIV-1 p24 antigen into cell supernatants was also inhibited by MG1 infection (Fig. 3E). Importantly, UV-inactivated MG1 had no effect on proviral HIV-1 DNA or p24 release, indicating that replication-competent oncolytic virus was required for the killing of HIV-infected MDM. Together, these data indicate that MG1 infection results in the preferential death of HIV-infected MDM.

### MG1-mediated killing of HIV-infected monocyte-derived macrophages is not mediated by soluble factors.

**FIG 4:**
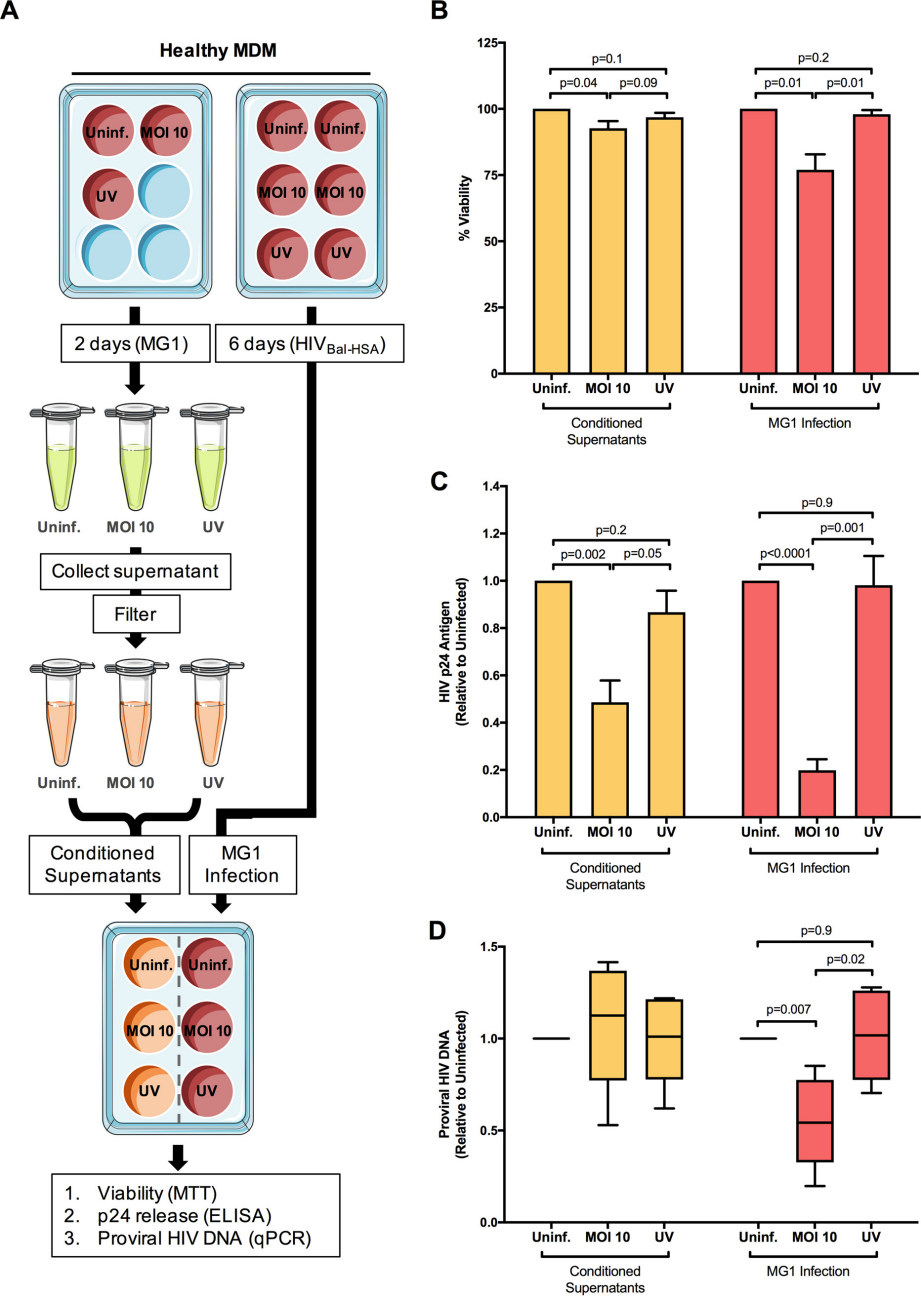
Conditioned supernatants from MG1-infected, HIV-1-negative MDM block p24 release without the preferential killing of HIV-infected MDM. (A) Supernatant transfer experimental workflow. MDM from healthy donors were plated in duplicate and either infected with MG1 (MOI of 10, UV-inactivated MG1 [UV], or uninfected [Uninf.]) or infected with HIV NL4.3 BAL-IRES-HSA. UV-inactivated viral particles were added to MDM cultures at a ratio of 10:1. Supernatants from MG1-infected MDM were collected at 2 dpi, filtered with Amicon ultra centrifugal filter units (MWCO, 100 kDa), and stored at −80°C. At 6 dpi, HIV-infected MDM cultures were infected with MG1 at an MOI of 10, left uninfected, or treated with UV-inactivated MG1. In duplicate, HIV-infected MDM were treated with filtered supernatants collected from autologous HIV-1-uninfected MDM. At 48 h post-MG1 infection, cell viability was assessed by the MTT assay, and proviral HIV-1 DNA was measured by qPCR. In certain experiments, cell supernatants were collected at 0, 2, 4, and 6 days post-MG1 infection for the measurement of HIV-1 p24 release by ELISA. (B) MDM viability, as measured by the MTT assay relative to the respective uninfected control, at 48 h post-MG1 infection or supernatant transfer. (C) HIV-1 p24 antigen in culture supernatants, relative to MG1-uninfected control, at 6 days post-MG1 infection or supernatant transfer. (D) Proviral HIV-1 DNA, measured relative to the respective uninfected control, at 48 h post-MG1 infection or supernatant transfer. *n* = 6; *P* values were calculated by a paired, two-tailed *t* test. Data represent mean ± SEM; *n* values represent separate biological replicates.

**FIG 5:**
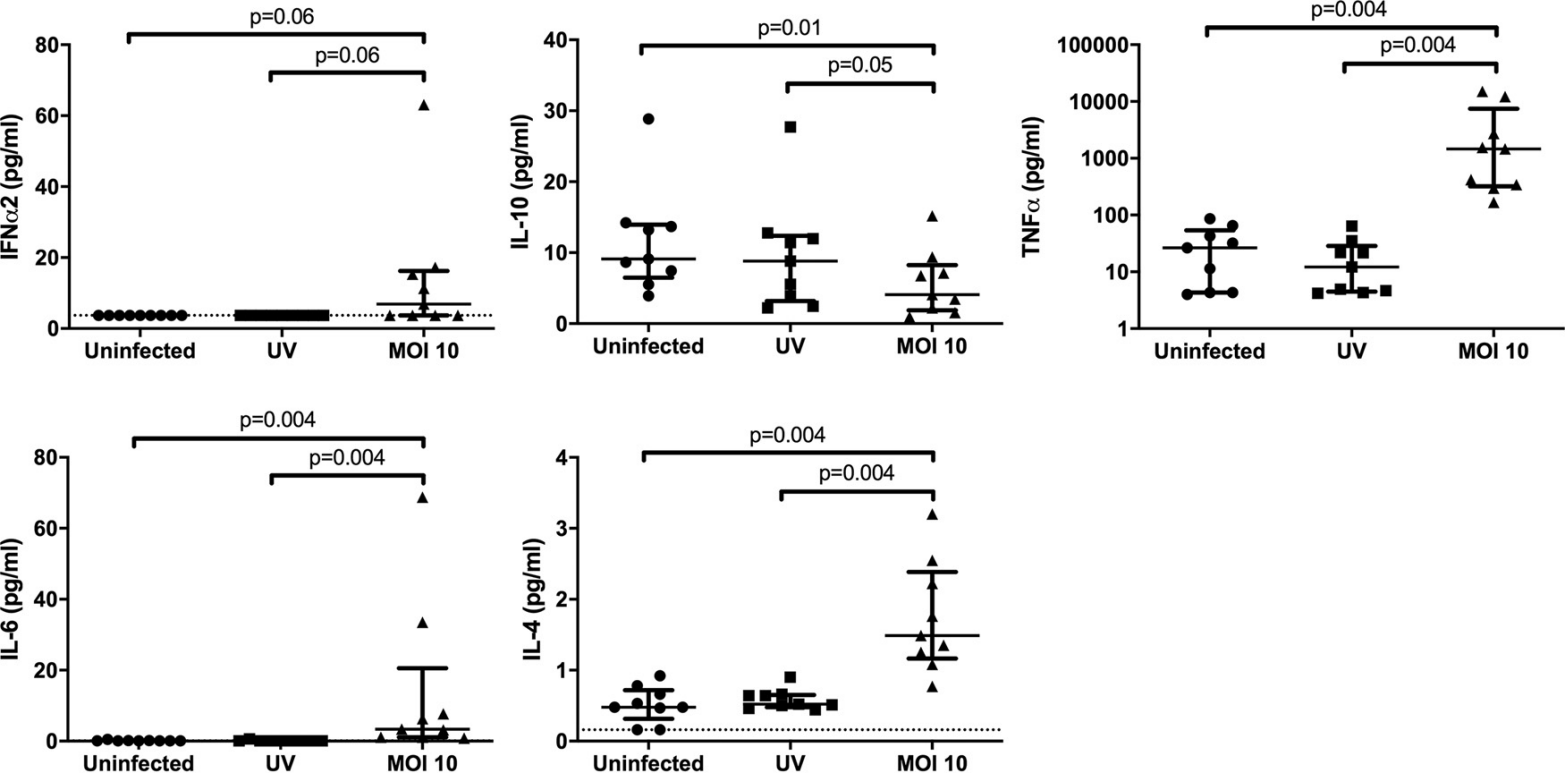
MG1 infection induces cytokine secretion by MDM. Supernatants were collected at 48 hpi from MG1-infected, HIV-uninfected MDM, and concentrations of IFN-α2, IL-10, TNF-α, IL-6, and IL-4 were measured via Luminex technology. *n* = 9; *P* values were calculated by Wilcoxon matched-pairs signed rank test (nonparametric), Data represent median with interquartile range; *n* values represent separate biological replicates.

Because cell death was observed in both GFP^+^/HSA^+^ and GFP^−^/HSA^+^ cells, we considered the possibility that MG1 infection was also causing the indirect, cytokine-mediated killing of HIV-infected cells. To address this, we performed supernatant transfer experiments, as depicted in Fig. 4A. Cells that received conditioned supernatants from MG1-infected MDM showed a small but significant drop in viability as measured by the MTT [3-(4,5-dimethyl-2-thiazolyl)-2,5-diphenyl-2H-tetrazolium bromide] assay (Fig. 4B). Both MG1 infection and conditioned supernatants also prevented the accumulation of HIV-1 p24 antigen in culture medium (Fig. 4C), but only MG1 infection resulted in a decrease in proviral HIV-1 DNA (Fig. 4D). Additionally, no effect on HIV-1 p24 release or proviral HIV-1 DNA was seen following exposure to either UV-inactivated MG1 or supernatants from MDM exposed to UV-inactivated MG1. Measurement of cytokines in filtered supernatants showed that concentrations of IFN-α2, tumor necrosis factor alpha (TNF-α), interleukin 4 (IL-4), and IL-6 were elevated at 48 h post-MG1 infection (Fig. 5). IFN-γ was also measured but was undetectable in all samples analyzed. In summary, the eradication of HIV-infected MDM is not mediated by soluble factors and requires the presence of infectious MG1.

### ISG induction is impaired within HIV-infected monocyte-derived macrophages.

**FIG 6:**
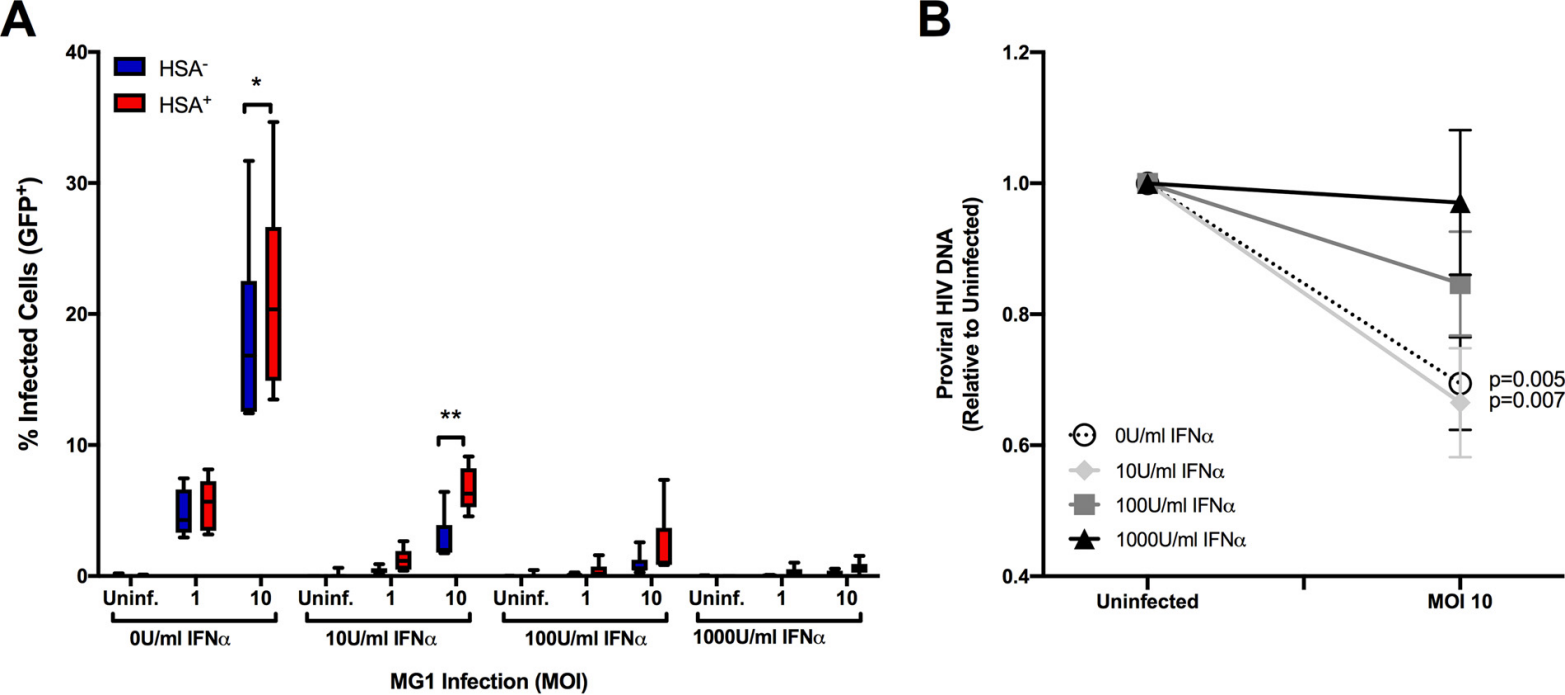
IFN-α protects HIV-infected and bystander MDM from MG1 infection and killing in a dose-dependent manner. (A) To assess the effect of IFN-I on MG1 infection of HSA^−^ and HSA^+^ MDM, cells were pretreated with increasing doses of IFN-α 24 h prior to MG1 infection. Frequencies of GFP^+^ MDM within HSA^−^ and HSA^+^ cell populations at 48h post-MG1 infection were then measured by flow cytometry (*n* = 6; *P* < 0.0001 by 2-way repeated-measures ANOVA; *, *P* < 0.05, **, *P* < 0.01 by Bonferroni posttest.). (B) Proviral HIV-1 DNA, measured relative to MG1-uninfected control, at 48 h post-MG1 infection. MDM were pretreated with IFN-α 24 h prior to MG1 infection (*n* = 7; *P* values were calculated by paired, two-tailed *t* test). Data represent mean ± SEM; *n* values represent separate biological replicates.

As MG1 is highly sensitive to IFN-I (17), we next wanted to determine if IFN-I signaling influenced OV-mediated cytopathogenicity. We began by assessing MG1 infection and killing in IFN-α-pretreated MDM cultures and found that stimulation with IFN-α blocked both MG1 infection (Fig. 6A) and the reduction of proviral HIV-1 DNA in a dose-dependent manner (Fig. 6B). Interestingly, preferential infection and the corresponding reduction in proviral HIV-1 DNA were maintained after treatment with low doses of IFN-α, leading us to investigate whether or not differences in ISG expression existed between HIV-infected and bystander cells.

**FIG 7:**
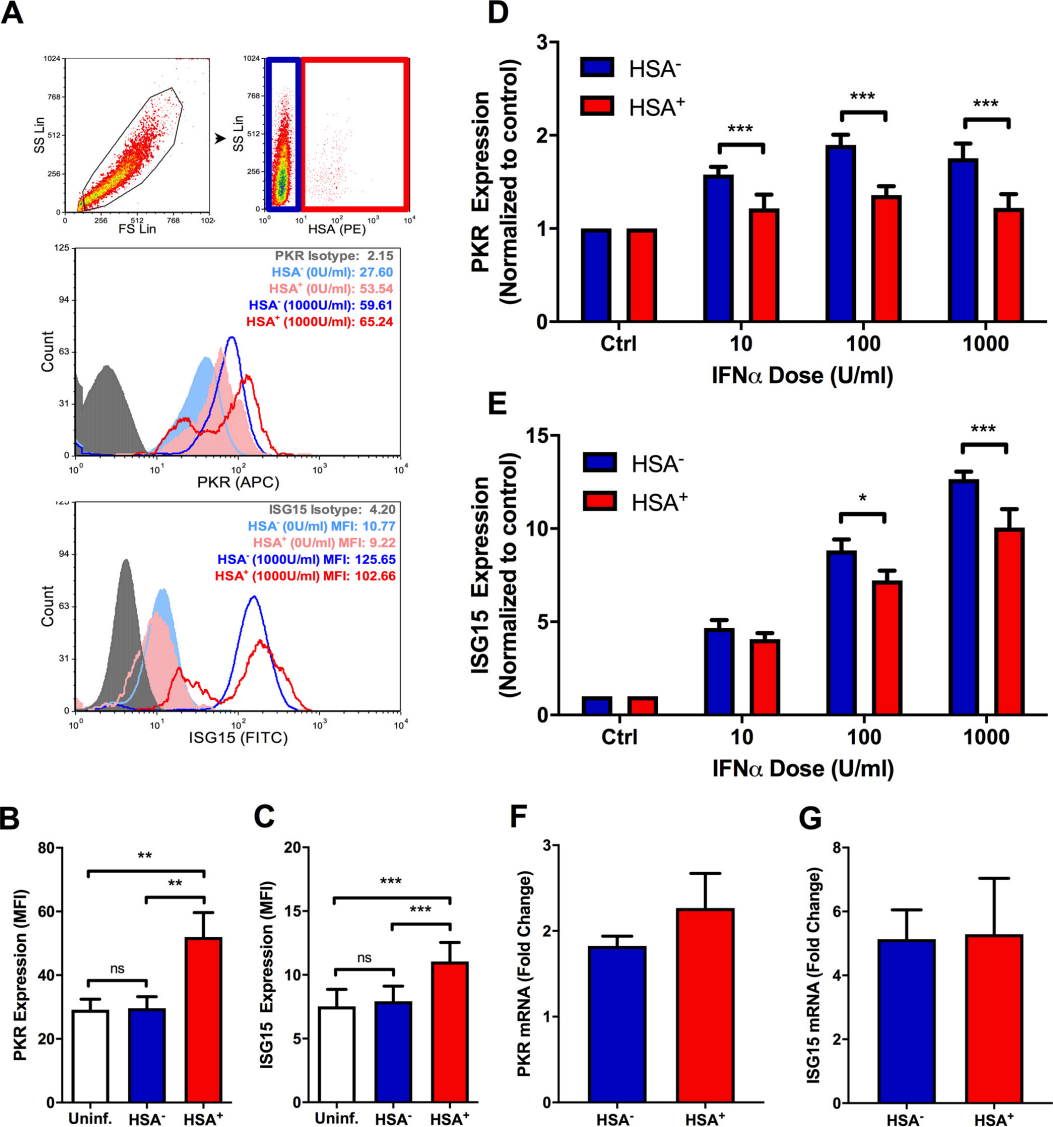
Differences in basal and IFN-α-induced ISG expression exist between HIV-infected and bystander MDM. (A) Flow cytometry gating strategy. Intact cells were gated (black), after which HSA^−^ (blue) and HSA^+^ (red) MDM were defined. Representative histograms show PKR (top) and ISG15 (bottom) induction in IFN-α-stimulated MDM. Respective isotype controls are shown in gray; HSA^−^ MDM are shown in blue (filled, unstimulated; open, stimulated); HSA^+^ MDM are shown in red (filled, unstimulated; open, stimulated). Histogram peak counts (*y* axis) for HSA^−^ and HSA^+^ populations were normalized to that of the isotype control for visualization purposes. SS Lin, side scatter, linear scale; FS Lin, forward scatter, linear scale. (B) Basal PKR expression in uninfected (white), HSA^−^ (blue), and HSA^+^ (red) MDM (*n* = 6; *P* = 0.001 by 1-way repeated measures ANOVA; **, *P* < 0.01 by Bonferroni posttest). (C) Basal ISG15 expression in uninfected (white), HSA^−^ (blue), and HSA^+^ (red) MDM (*n* = 7; *P* = 0.0001 by 1-way repeated measures ANOVA; ***, *P* < 0.001 by Bonferroni posttest). (D) Relative PKR induction following 24 h of IFN-α stimulation, normalized to respective unstimulated controls (*n* = 6; *P* = 0.023 by 2-way repeated measures ANOVA; ***, *P* < 0.001 by Bonferroni posttest.). (E) Relative ISG15 induction following 24 h of IFN-α stimulation, normalized to respective unstimulated controls (*n* = 7; *P* < 0.0001 by 2-way repeated measures ANOVA, *, *P* < 0.05, ***, *P* < 0.001 by Bonferroni posttest; ns, not significant). (F) PKR mRNA measured in HSA^+^ and HSA^−^ MDM at 16 h poststimulation with 1,000 U/ml IFN-α. (G) ISG15 mRNA measured in HSA^+^ and HSA^−^ MDM at 16 h poststimulation with 1,000 U/ml IFN-α. ΔΔ*CT*s used to determine fold change were calculated by normalizing threshold cycle (*C_T_*) values to those of the respective untreated control and GAPDH (*n* = 4). Data represent mean ± SEM; *n* values represent separate biological replicates.

**FIG 8:**
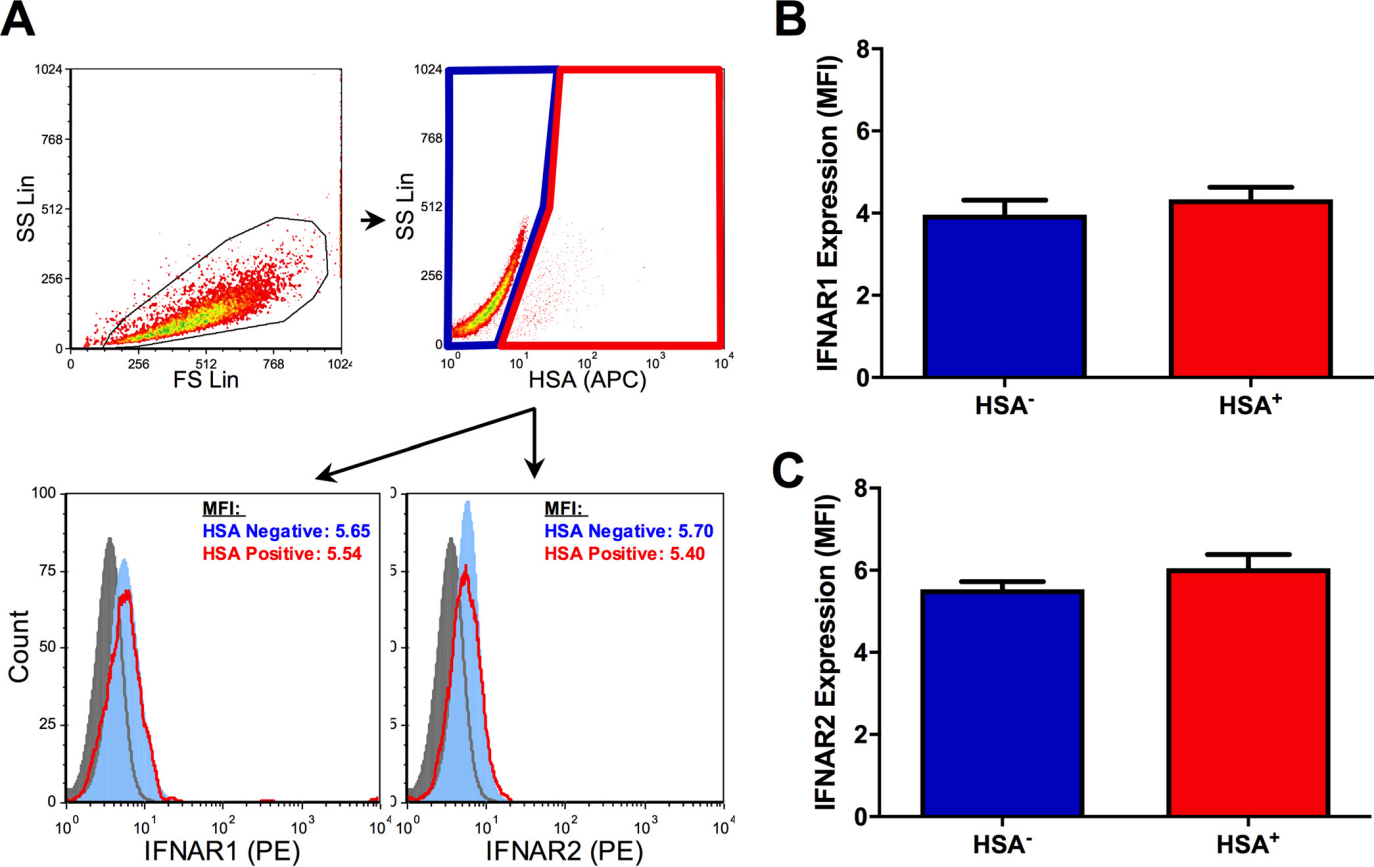
Surface expression of IFNAR1/2 on HSA^+^ and HSA^−^ MDM. (A) Flow cytometry gating strategy and representative histograms (filled gray, PE FMO control; filled light blue, HSA^−^ MDM; empty red, HSA^+^ MDM) depicting IFNAR1 and IFNAR2 expression on HSA^−^ and HSA^+^ MDM. Intact cells were gated (black), after which HSA^−^ (blue) and HSA^+^ (red) MDM were defined. Histogram peak counts (*y* axis) for HSA^−^ and HSA^+^ populations were normalized to that of the PE FMO control for visualization purposes. (B) IFNAR1 expression on HSA^−^ (blue) and HSA^+^ (red) MDM, as measured by mean fluorescent intensity (*n* = 7). (C) IFNAR2 expression on HSA^−^ and HSA^+^ MDM, as measured by mean fluorescent intensity (*n* = 7). Data represent mean ± SEM; *n* values represent separate biological replicates.

To do this, HIV-1-infected MDM cultures were stimulated with IFN-α, and levels of two representative ISG products, PKR and ISG15, were measured by flow cytometry (Fig. 7A). Basal expression of both proteins was higher in HSA^+^ MDM than in both HSA^−^ MDM and HIV-1 naive MDM (Fig. 7B and C). Conversely, the relative IFN-α-induced expression of PKR and ISG15 was lower in HSA^+^ MDM (Fig. 7D and E), indicating impaired IFN-α responsiveness in HIV-infected cells. This was not associated with differences in ISG15 or PKR mRNA levels in sorted HSA^+^ and HSA^−^ MDM (Fig. 7F and G). Additionally, surface expression of the IFN-α/β receptor (subunits 1 and 2; IFNAR1/2) did not differ between HSA^+^ and HSA^−^ cells (Fig. 8), suggesting that differences in ISG induction in these cell populations was not due to receptor downregulation.

### MG1 eliminates HIV-infected alveolar macrophages *ex vivo*.

Finally, to begin to address the potential clinical implications of our findings, we investigated whether MG1-mediated killing of HIV-infected myeloid cells could be replicated in primary alveolar macrophages. As one of the only accessible sources of tissue-resident myeloid cells, AM are known to harbor replication-competent HIV-1 and thus act as a viral reservoir (2, 20–22, 35). To assess whether HIV-infected AM were susceptible to MG1-mediated killing, cells were collected from PLWHIV (on suppressive ART for ≥3 years at the time of collection) via bronchoalveolar lavage, isolated by plate adherence, and infected with MG1.

**FIG 9:**
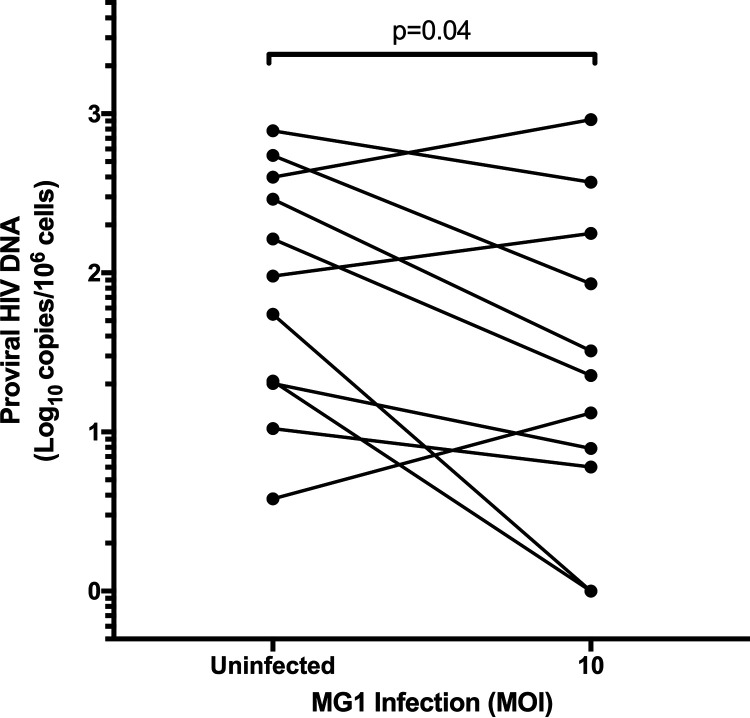
MG1 infection reduces proviral HIV-1 DNA in alveolar macrophages from PLWHIV. Alveolar macrophages were allowed to adhere for 2 h at 37°C following collection by bronchoalveolar lavage. Subsequently, nonadherent cells were removed by washing, and adherent alveolar macrophages were infected with MG1 at an MOI of 10. Cell pellets were collected at 48 h postinfection, and proviral HIV DNA was measured by ddPCR. *n* = 11 (representative of separate biological replicates); *P* value calculated by paired, two-tailed *t* test.

Following MG1 infection of adherent AM, proviral HIV-1 DNA was measured by digital droplet PCR (ddPCR). As has been previously demonstrated using cells collected from the lungs of PLWHIV (35), the frequency of HIV-infected AM was highly variable. Despite a relatively small number of participants, a statistically significant decrease in proviral HIV-1 DNA was observed at 48 h post-MG1 infection (Fig. 9), emphasizing the consistency of this effect. Additionally, MG1 infection did not appreciably reduce CD3 DNA copy number (as quantified by ddPCR) in comparison to MG1-uninfected AM, suggesting that HIV-uninfected AM were not killed by MG1. As primary, tissue-resident macrophages have been historically overlooked in HIV-1 cure research (reviewed in references 36), these data provide important insights into the future application of MG1 as an HIV-1 cure strategy.

## DISCUSSION

The development of an HIV-1 cure has focused largely on the identification, characterization, and eradication of latently infected CD4^+^ T cells. Unfortunately, many of these strategies remain untested in other cellular reservoirs of HIV-1. We therefore sought to identify whether MG1, an established oncolytic rhabdovirus capable of targeting latently HIV-infected cell lines and primary CD4^+^ T cells (19), could also infect and kill HIV-infected macrophages.

In agreement with our previous findings in CD4^+^ T cells, we observed a higher frequency of MG1 infection in HSA^+^ (HIV-infected) MDM than in HSA^−^ (bystander) MDM. This coincided with the preferential killing of HIV-infected MDM, as measured by an increase in annexin V^+^ cells and a reduction in proviral HIV-1 DNA. Next, we investigated whether MG1-mediated killing of HIV-infected MDM could be achieved via indirect means. UV-inactivated viral particles, for instance, have been found to induce the cytolytic killing of acute myeloid leukemia cells *in vitro* and in a murine model of leukemic blast crisis (37). OV-induced release of proinflammatory cytokines may also facilitate the indirect killing of tumor cells (38). In the context of HIV-1 infection, however, UV-inactivated MG1 did not kill HIV-infected MDM (Fig. 3D) or stimulate the release of proinflammatory cytokines such as TNF-α or IL-6 (Fig. 5).

Similarly, treatment of HIV-infected cells with conditioned supernatants from MG1-infected MDM did not eliminate HIV-infected cells but resulted in a significant reduction in HIV-1 p24 antigen release (Fig. 4C and D). IFN-α2, TNF-α, IL-4, and IL-6 were elevated in supernatants from MG1-infected cells, which is consistent with previous literature describing cytokine release by cells infected with MG1 or with the closely related rhabdovirus, vesicular stomatitis virus (31, 39, 40). Cytokines including TNF-α, IL-6, and IFN-γ have also been shown to block HIV replication in human macrophages (41–44), suggesting that these soluble factors are responsible for the reduction in p24 release following supernatant transfer. Given that the eradication of HIV-infected MDM requires the presence of replication-competent MG1, avoiding off-target effects by enhancing the selectivity of MG1 for HIV-infected cells remains an important objective that is under investigation by our group. Nonetheless, healthy cynomolgus macaques were found to tolerate MG1 doses of 1,011 PFU, providing promise for the ongoing investigation of OV as an HIV-1 cure strategy (45).

Next, we investigated the role of IFN-I signaling in MG1 infection. The MG1 virus is highly sensitive to IFN-I signaling due to two genetically engineered amino acid substitution mutations within the G (Q242R) and M (L123W) proteins (17). It was therefore no surprise that IFN-α pretreatment protected both HSA^+^ and HSA^−^ MDM from MG1 infection in a dose-dependent manner (Fig. 6).

To investigate whether we could identify global IFN-I signaling differences between HSA^+^ and HSA^−^ MDM, we measured the basal and IFN-α-induced expression of two representative ISG. The observed baseline elevation of PKR and ISG15 in HSA^+^ MDM was as expected, given that ISG induction has been shown to occur within the first 24 h of HIV-1 infection in MDM (46). Nonetheless, it was interesting to see that higher levels of ISG were not sufficient to block preferential MG1-mediated cytopathogenicity, as elevated ISG expression in malignant cells has been found to be protective against OV infection and killing (47). One possible explanation is the functional impairment of ISG in HIV-infected MDM. The interaction of PKR with a number of cellular and viral proteins in HIV-1-infected cells has already been shown to block this ISG from fulfilling its role as a key RNA sensing protein needed for the activation of cellular antiviral response pathways (48–51). Additionally, given that pretreatment with low levels of IFN-α did not prevent MG1-mediated killing of HIV-infected cells (Fig. 6B), and that HIV-infected MDM are impaired in their ability to produce IFN-I (14), it is possible that additional defects in antiviral signaling that sensitize these cells to OV infection exist.

As evidence of altered IFN-I signaling in HIV-infected MDM, we also showed that the IFN-α-mediated induction of ISG15 and PKR was impaired in HSA^+^ MDM. This is consistent with findings previously reported by Ranganath et al. in latently HIV-infected, pro-myeloid OM10.1 cells (18). IFN-α/β receptor downregulation (52, 53) and impaired STAT1/2 activation (10, 54, 55) have been found to prevent IFN-I-induced ISG mRNA expression in the context of HIV-1 infection. No differences in IFNAR1/2 surface expression or PKR and ISG15 mRNA were observed between HSA^+^ and HSA^−^ MDM, however, suggesting a posttranscriptional mechanism of impairment. In support of this hypothesis, HIV-1-mediated inhibition of cellular translational machinery has been previously reported (56–59). The small number of HSA^+^ MDM obtained via magnetic bead separation, as well as the fragility of these cells postisolation, was an experimental limitation that unfortunately prevented the further study of translational defects in HIV-infected cells. Nonetheless, these data remain unique in their use of a reporter virus model to study and compare IFN-I signaling in HIV-infected and bystander MDM populations.

Finally, we demonstrated that MG1 could eliminate HIV-infected macrophages *ex vivo*, using AM from ART-treated PLWHIV. AM form a unique source of accessible, tissue-resident myeloid cells that form a known HIV-1 reservoir (60). The ability to eradicate these cells using an IFN-I-sensitive OV therefore has important implications for the development of an HIV-1 cure. Low cell numbers, and the relative fragility of cells obtained by bronchoalveolar lavage, unfortunately prevented further investigation of markers of MG1 infection, cell death, and IFN-I signaling, as was possible with *in vitro* HIV-infected MDM. This, combined with the relatively small number of donors recruited for bronchoalveolar lavage and AM collection, represents a current limitation of this study objective. Still, the decision to assess MG1-mediated elimination of HIV-infected cells using primary AM from PLWHIV represents an important step forward in the field of HIV-1 cure research. Primary, tissue-resident macrophages have consistently been overlooked as a source of replication-competent HIV-1 and, consequently, are a necessary target for novel HIV-1 cure strategies. Future work assessing the efficacy of latency reversal agents, allogeneic stem cell transplant, or other gene therapy approaches on HIV-1 reservoir size must therefore consider the myeloid HIV-1 reservoir as part of the experimental design (36).

In summary, these results demonstrate the preferential infection and killing of HIV-infected MDM by the IFN-I-sensitive oncolytic virus MG1. In doing so, we have fulfilled a current objective in HIV-1 cure research, which is to consider and account for myeloid HIV-1 reservoirs when testing new therapeutic strategies that have already been investigated in CD4^+^ T cells (36). Going forward, the ability of MG1 to reduce the size of the HIV-1 reservoir will need to be further assessed using a relevant *in vivo* model of HIV-1 infection. Should ongoing phase I/II clinical trials of MG1 in cancer (ClinicalTrials registration numbers NCT02285816, NCT02879760, and NCT03618953) identify this oncolytic virus as safe, a proof-of-concept clinical trial to investigate the safety and impact on HIV reservoir size in PLWHIV may be feasible.

## MATERIALS AND METHODS

### Reagents.

Gibco RPMI 1640, Gibco Dulbecco’s modified Eagle medium (DMEM), and Gibco phosphate-buffered saline (PBS), pH 7.4, were purchased from Life Technologies (Carlsbad, CA). Gibco Opti-MEM reduced serum medium was purchased from ThermoFisher Scientific. Additional reagents include heat-inactivated fetal bovine serum (FBS), normal goat serum (NGS), penicillin (100 U/ml) and streptomycin (100 µg/ml) (PenStrep), and l-glutamine (all from Life Technologies), heat-inactivated human AB serum (Valley Biomedical, Winchester, VA), universal type I alpha interferon (IFN-α) (PBL Assay Science, Piscataway, NJ), recombinant human macrophage colony-stimulating factor (M-CSF; carrier-free) (BioLegend catalog no. 574802), maraviroc (NIH AIDS Reagent Program, Division of AIDS, NIAID, NIH; catalog no. 11580), and Lipofectamine 2000 transfection reagent (Invitrogen, Burlington, ON, Canada).

### Ethics statement.

Experiments requiring healthy volunteers were approved by the Ottawa Health Science Network Research Ethics Board (protocol no. 2005388-01H), and all participants provided written informed consent. The collection and use of alveolar macrophages from PLWHIV were approved by the Institutional Review Boards of the MUHC (no. 15-031) and the Université du Québec à Montréal (no. 602). All study participants provided written informed consent.

### Cell culture.

Vero (CCL-81) and HEK293T cells (CRL-3216) were obtained from the American Type Culture Collection (Manassas, VA) and cultured in DMEM with 10% fetal calf serum (FCS) and PenStrep. ACH-2 cells were obtained via the NIH AIDS Reagent Program, Division of AIDS, NIAID, NIH, from Thomas Folks (61, 62) and cultured in RPMI 1640 supplemented with 10% FCS, PenStrep, and l-glutamine (2 mM). Cells were maintained at 0.2 × 10^5^ to 1 × 10^6^ cells/ml by passaging every 2 to 3 days.

Monocytes were separated from healthy donor peripheral blood mononuclear cells (PBMC) by plate adherence. Following isolation by density gradient centrifugation, PBMC were resuspended at 6.25 × 10^6^/ml in warm, serum-free RMPI 1640 with PenStrep. A total of 1.25 × 10^8^ PBMC were then plated in 150-cm^2^ polystyrene tissue culture dishes (Sarstedt, Nümbrecht, Germany) and left to adhere for 2 h at 37°C. Plates were washed 3 times with endotoxin-free PBS (pH 7.4; Gibco) to remove nonadherent lymphocytes, and 20 ml of warmed RPMI 1640, supplemented with PenStrep and 10% heat-inactivated human AB serum (Mϕ medium) and M-CSF (25 U/ml), was added to the plate. Adherent cells were incubated at 37°C with 5% CO_2_ for 7 days. At 3 days postplating, cells were washed twice with warmed endotoxin-free PBS, and 20 ml of Mϕ medium was added to the plate. On day 8, adherent MDM were washed twice with endotoxin-free PBS, detached using Accutase (Millipore-Sigma) and gentle scraping with a Sarstedt cell scraper, and counted by trypan blue exclusion. MDM were then pelleted by centrifugation (300 × *g* for 10 min), resuspended at 2.5 × 10^5^ cells/ml in Mϕ medium, and plated in either 6-well (5 × 10^5^ cells/well) or 12-well (2.5 × 10^5^ cells/well) plates for further experiments.

AM were isolated from bronchoalveolar lavage fluid by plate adherence, as described previously (35, 63, 64). Briefly, participants recruited at the McGill University Health Centre (MUHC, Montreal, Canada) were cART-treated PLWHIV, with suppressed plasma viral load for ≥3 years and without respiratory symptoms or active illness. A total of 50 to 100 ml of lavage fluid was collected during bronchoscopy. Cells were pelleted and washed at 180 × *g* for 10 min and then counted by trypan blue exclusion. Cells were then resuspended in serum-free RPMI 1640 at 5 × 10^5^ cells/ml and plated in 24-well plates at 2.5 × 10^5^ cells/well for 2 h at 37°C. Nonadherent cells were removed by rinsing the wells with endotoxin-free PBS, and adherent cell were covered with 500 µl of Mϕ medium. Prior to collection, adherent AM were rinsed an additional 3 times using endotoxin-free PBS, in order to remove cellular debris and nonadherent lymphocytic cells. AM were detached at 37°C for 30 min using CellStripper dissociation reagent (Corning, Fisher Scientific), followed by gentle pipetting.

### Production of virus stocks.

The HIV NL4.3 BAL-IRES-HSA plasmid was obtained from Michel J. Tremblay at Université Laval. HEK293T cells were seeded at 2 × 10^6^ cells/T75 flask and transfected with 20 µg of purified plasmid using Lipofectamine 2000 and Opti-MEM I reduced serum medium, in accordance with the manufacturer’s instructions. Supernatants were collected at 48 h posttransfection and filtered through 0.45-µm and 0.22-µm polyvinylidene fluoride (PVDF) filters (UltiDent Scientific, St. Laurent, QC, Canada). HIV-1 p24 concentration was quantified by ELISA.

The GFP-expressing recombinant OV MG1 (obtained from John Bell and David Stojdl) was propagated and titered on Vero cells, as described previously (17, 65). UV inactivation of MG1 stocks was performed as described previously (19).

### *In vitro* HIV-1 infection and enrichment of HSA^+^ MDM.

MDM were infected with HIV NL4.3 BAL-IRES-HSA for 6 days at 37°C with 5% CO_2_. HIV-1 infection was confirmed by quantitative PCR (qPCR) (66), p24 ELISA, and surface expression of virus-encoded murine heat-stable antigen (HSA) by flow cytometry (24). HSA-expressing MDM were isolated by positive selection using Miltenyi LS columns in combination with the MidiMACS magnet (Miltenyi Biotech). The HSA sorting protocol was optimized by the laboratory of Michel Tremblay, as described previously (24, 25). Purity of the HSA^+^ and HSA^−^ fractions was assessed by flow cytometry.

### *In vitro* MG1 infection and supernatant transfer experiments.

MDM and AM were infected with MG1 in RPMI 1640, supplemented with PenStrep and 10% heat-inactivated human AB serum (Mϕ medium), with 10 µM maraviroc. Cells pellets were collected at 48 hpi and stored at −80°C for quantification of HIV-1 DNA. MG1 infection, frequency of HSA-expressing cells, and cell viability were assessed in MDM cultures by flow cytometry and MTT assay.

For supernatant transfer experiments, MDM from healthy donors were infected with either MG1 or HIV NL4.3 BAL-IRES-HSA. At 48 hpi, supernatants from MG1-infected cells were collected, filtered with Amicon Ultra centrifugal filter units (molecular weight cutoff [MWCO], 100 kDa; Millipore Sigma) and stored at −80°C. Removal of infectious MG1 was confirmed on Vero cells by flow cytometry. At 6 dpi, HIV-infected MDM cultures were infected with MG1 at an MOI of 10, left uninfected, or treated with UV-inactivated MG1. In duplicate, HIV-infected MDM were treated with filtered supernatants, at a 1:1 ratio with fresh Mϕ medium. At 48 h post-MG1 infection, cell viability was assessed by MTT assay and proviral HIV-1 DNA was measured by qPCR. Cell-free supernatants were collected every 2 days for quantification of HIV-1 p24 antigen by ELISA. MDM were pelleted at 6 dpi and stored at −80°C for quantification of proviral DNA.

### Measurement of supernatant cytokines.

IFN-α2, IFN-γ, TNF-α, IL-4, IL-6, and IL-10 were measured in filtered supernatants collected at 2 dpi from MG1-infected, HIV-uninfected MDM using the Milliplex human cytokine/chemokine/growth factor panel A kit (catalog no. HCYTA-60K-06; Millipore). The panel was read using Luminex technology on a MAGPIX system (Luminex xPONENT for MAGPIX version 4.2; Luminex Corporation, Austin, TX), Analysis was performed via Milliplex Analyst version 5.1.0.0 software (Vigenetech Inc., Carlisle, MA).

### IFN-α stimulation of MDM.

MDM were stimulated with IFN-α at 6 days post-HIV-1 infection. IFN-α was diluted in Mϕ medium and added directly to the wells, after which MDM were incubated at 37°C for 16 h for mRNA isolation or 24 h for flow cytometry analysis.

### Flow cytometry.

Analysis was performed using the FC500 Beckman Coulter flow cytometer (Beckman Coulter) and FCS Express Research edition 4.0 (De Novo Software, Los Angeles, CA). In preparation for staining, MDM were detached with Accutase and gentle pipetting, aliquoted into polypropylene tubes (1 × 10^5^ cells/tube), and washed with PBS–1% BSA (460 × *g* for 5 min).

For HSA staining, cells were resuspended in cold PBS plus 10% human AB serum and 20% NGS and incubated at 4°C for 20 min. The anti-HSA antibody (clone M1/69; BioLegend) or HSA isotype control antibody (rat IgG2b κ isotype clone RTK4530; BioLegend) was then added at 3 µg/ml, and cells were incubated at 4°C for 15 min. Cells were then washed twice and prepared for either further staining or for sorting.

Expression of the IFN-α/β receptor subunit 1 (R&D Systems; clone no. 85228), the IFN-α/β receptor subunit 2 (Miltenyi; clone REA124), and the LDL receptor (LDLR) (R&D Systems; clone no. 472413) was measured by blocking MDM at 4°C for 15 min in PBS–1% BSA with 10 µl of FcR blocking reagent (Miltenyi Biotec). Cells were then stained at 4°C for 30 min in 100 µl of PBS–1% BSA plus 10 µl of the antibody of interest. Prior to analysis, cells were washed and fixed in 1% paraformaldehyde (PFA). Fluorescence minus one (FMO) controls, in which cells were stained only with the allophycocyanin (APC)-conjugated anti-HSA antibody, were used as the negative control.

Annexin V staining was performed using the eBioscience phycoerythrin (PE)/Cy7 annexin V detection kit (ThermoFisher Scientific), by following the manufacturer’s instructions. Cells were stained with 5 µl of annexin V for 15 min at 4°C, washed twice with 1× annexin V binding buffer (ThermoFisher Scientific), and then fixed, as described above.

ISG15 staining was performed on PFA-fixed MDM using 5 μl of the Alexa Fluor 488-conjugated anti-human ISG15 (R&D Systems; clone no. 851701) or 5 μl of the isotype-matched control (R&D Systems; clone no. 54447). PKR staining was performed on PFA-fixed MDM using 1 μl unconjugated mouse anti-human PKR antibody (Abcam; clone 6H3A10), followed by staining with 4 μl of the APC-conjugated goat anti-mouse IgG (Abcam) or 2 μl isotype-matched control (1 mg/ml) (Abcam; clone 1F8). All incubations were performed at 4°C for 30 min, in 100 μl of 0.5% saponin with 10% NGS. Cells were washed and resuspended in PBS–1% BSA for analysis.

### MTT assay.

Cell viability was assessed by using a Vybrant MTT cell proliferation assay kit (Invitrogen), in accordance with the manufacturer’s instructions. At 2 days post-MG1 infection, the MTT [3-(4,5-dimethylthiazol-2-yl)-2,5-diphenyltetrazolium bromide] stock solution was diluted 1:15 with phenol red-free RPMI 1640 (supplemented with PenStrep and 10% FCS) and added to MDM. Cells were incubated for 2 h at 37°C, at which point viral particles were lysed for 1 h at 37°C using a 10% sodium dodecyl sulfate (SDS; Fisher Scientific) solution containing 0.01 M HCl (Fisher Scientific). Absorbance was read at 570 nm using a Multiskan Ascent 96 plate reader (MTX Lab Systems Inc., Bradenton, FL).

### p24 ELISA.

The HIV-1 p24 antigen capture kit (Frederick National Laboratory for Cancer Research, Frederick, MD; NIH AIDS Reagent Program) was used to quantify HIV-1 p24 antigen in cell-free supernatants, as described previously (19).

### DNA and RNA extraction and quantification.

Genomic DNA extraction and quantification of proviral HIV-1 DNA were performed using a previously described protocol and primer-probe set (66). Detection of proviral HIV-1 DNA in AM was performed using digital droplet PCR (ddPCR), as described previously (19), and absolute quantification of proviral HIV-1 DNA and CD3 DNA used the QX200 droplet reader (Bio-Rad) and QuantaSoft software version 1.7 (Bio-Rad).

Cell-associated RNA was extracted using the Illustra RNAspin minikit (GE Healthcare Life Sciences, Mississauga, ON, Canada) in accordance with the manufacturer’s instructions. RNA integrity was monitored by agarose gel electrophoresis, and concentrations were measured using an ND-1000 spectrophotometer (NanoDrop, Wilmington, DE). RNA was converted to cDNA using iScript reverse transcription supermix for reverse transcription (RT)-qPCR (Bio-Rad). ISG15, PKR, and GAPDH (glyceraldehyde-3-phosphate dehydrogenase) cDNA was then detected using Bio-Rad’s PrimePCR SYBR green assay (ISG15 assay no. qHsaCED0001967; PKR assay no. qHsaCED0042156; GAPDH assay no. qHsaCED0038674), using the CFX Connect real-time PCR detection system (Bio-Rad). Amplicon purity was confirmed using a 1.5% agarose gel. RNA and cDNA were stored at –80°C in single-use aliquots.

### Artwork.

Figure 4A was created using Smart Servier Medical Art image sets. Servier Medical Art by Servier is licensed under a Creative Commons Attribution 3.0 Unported License.

### Statistics.

Statistics were performed using GraphPad Prism 5.0 software (San Diego, CA), and *P* values of ≤0.05 were considered significant. As determined *a priori*, the following statistical analyses were used. PKR and ISG15 expression between untreated and IFN-α-stimulated MDM, cell viability via MTT assay, HIV-1 p24 antigen release, proviral HIV-1 DNA, HIV-1 proviral DNA in MG1-infected AM, and surface expression of LDL-R were analyzed by a paired, two-tailed *t* test. IFN-α-induced PKR and ISG15 expression, MG1 infection in MDM at 48 hpi, MG1-induced cell death, and HIV-1 p24 antigen release were analyzed by 2-way repeated-measures analysis of variance (ANOVA) with the Bonferroni posttest. Proviral HIV-1 DNA at 48 hpi MG1 infection and basal ISG expression between uninfected, HSA^−^, and HSA^+^ MDM was analyzed by 1-way repeated-measures ANOVA with the Bonferroni posttest. MG1-induced cytokine secretion was analyzed by the Wilcoxon matched-pairs signed rank test (nonparametric).
